# Health and Dietary Disparities in the North of England: A Report From Nutrition North on Key Challenges in the North of England

**DOI:** 10.1111/nbu.70063

**Published:** 2026-07-31

**Authors:** Iain Brownlee, Julie Abayomi, Ruth Boocock, Emma Boyland, Heather Brown, Nicola Calder, Amy Finlay, Mark Green, Scott Lloyd, Amelia A. Lake, Sarah Hill, Bernard M. Corfe

**Affiliations:** ^1^ Faculty of Science and Environment Northumbria University Newcastle upon Tyne UK; ^2^ Faculty of Health, Social Care & Medicine Edge Hill University Ormskirk UK; ^3^ School of Health and Life Sciences Teesside University Middlesbrough UK; ^4^ Fuse, The Centre for Translational Research in Public Health Newcastle upon Tyne UK; ^5^ Faculty of Health and Life Sciences University of Liverpool Liverpool UK; ^6^ Division of Health Research Lancaster University Lancaster UK; ^7^ Food Active Programme, Health Equalities Group Liverpool UK; ^8^ Faculty of Science and Engineering University of Liverpool Liverpool UK; ^9^ Middlesbrough Council Middlesbrough UK; ^10^ Northern Health Science Alliance Droylsden Manchester UK; ^11^ Human Nutrition and Exercise Research Centre Newcastle University Newcastle upon Tyne UK

## 1 Background

Geographically and administratively, the North of England is defined as the area bounded by the three northernmost governmental regions—the North West, North East and Yorkshire and the Humber (Office for National Statistics 2021a). The region covers an area of around 40 million km^2^ (Office for National Statistics 2022) and includes nearly 16 million of the 57 million people in England (Office for National Statistics 2023b). While this News and Views article focuses on ‘The North’, the authors acknowledge that there is no singular and homogenous population of this area, with many previous reports highlighting heterogeneity at different sub‐levels (Office for National Statistics 2021b, 2021c, 2021d). A map and data presenting the geographical regions of England are available elsewhere (Office for National Statistics 2025).

From a geographical context, deprivation is believed to be a major factor driving people in the North to live shorter lives with increased burden of disease and lower economic potential than those in other regions of England (Munford et al. 2023). The latest findings from the National Diet and Nutrition Survey suggest that food group/nutrient intake (vs. recommendations) and nutrient status tend to be worse in UK adults and children from the most deprived backgrounds (Office for Health Improvement and Disparities 2025).

Several recent reports on health outcomes have highlighted that the North has poorer outcomes than other English regions, or indeed those in the wider UK (Health Equity North 2023; Office for Health Improvement and Disparities 2021a, 2021b, 2021c). Poor diet is believed to be the major, modifiable risk for avoidable morbidity and mortality globally (Afshin et al. 2019). There is scope to improve the dietary habits of almost everyone in England (Scheelbeek et al. 2020), although this may be particularly challenging in those with food access issues (Afshin et al. 2014).

Nutrition North is the latest expert group brought together by the Northern Health Sciences Alliance (NHSA). Nutrition North is a coalition of nutrition scientists, advocates, practitioners and industry professionals across the North of England, which aims to define better and to address the nutritional component of health and societal challenges faced in The North of England through:

1. Food and nutrition advocacy to improve diet and health in the region.
2. Supporting clinical nutrition delivery in The North.
3. Driving better collaboration between academic experts and the food and nutraceutical sectors regionally.
4. Linking with other expert groups supported by NHSA.

In order to progress these Aims, the Nutrition North Executive recently published a white paper report (Lake et al. 2025), entitled ‘Food, Health and Nutrition in the North of England: Inequalities & opportunities’. We aimed to use this report to define the state of nutrition in the North of England versus the national average, particularly in terms of dietary habits, health outcomes, food security and the food environment.

## 2 Key Findings of the Report

### 2.1 The North Has Worse Outcomes of Diet‐Related, Preventable Disease

Mortality rates in The North are the highest across regions in England, with over 100 additional deaths per 100 000 people compared to the national average. All three northern regions have the highest mortality rates in the country for preventable cancer; for preventable cardiovascular disease; for preventable liver disease (Office for Health Improvement and Disparities 2021a, 2021b, 2021c, 2024).

Adult and child obesity prevalence is higher in all regions of the North than average rates in England (Office for Health Improvement and Disparities 2021a, 2021b, 2021c, 2024). The UK has among the highest prevalence of overweight and obesity in adults and children of countries in Europe and the rest of the world (World Health Organization 2022; World Obesity Federation 2024). At a national level, there appears to be a much higher proportion of children defined as obese and overweight by the time they leave primary school compared to when they started (NHS England 2024). Within the North of England, prevalence of obesity (including severe obesity) at Reception (range 9.9%–11.3% vs. national average = 9.2%), Year 6 (19.4%–25.5% vs. 22.7%) and in adults (27.5%–32.4% vs. 25.9%) is worse than all other regions of the UK (Office for Health Improvement and Disparities 2024).

Cardiovascular diseases are the most frequent cause of death in the UK (Office for National Statistics 2023a) and have also been highlighted as the major cause of diet‐related disease globally (Afshin et al. 2019), particularly in relation to excess sodium intake and low intake of whole grains and other food sources of dietary fibre. The number of people with hypertension in the North East (16.6% of the adult population) is the highest in the country (Office for Health Improvement and Disparities 2021a, 2021b, 2021c, 2024). Prevalence in the North West (15.0%) and Yorkshire and the Humber (14.9%) is closer to the national average in England (14.4%) (Office for Health Improvement and Disparities 2024).

Diabetes is noted to be particularly problematic in terms of long‐term management of associated health issues (Diabetes UK 2019) management of diabetes is estimated to account for approximately 10% of total NHS England budget (National Health Service 2019). Prevalence is suggested to be particularly high in South Asian and Caribbean or African communities in the UK (Goff 2019), including those in the North of England. Diabetes prevalence in all three regions of the North is higher than the national average (Office for Health Improvement and Disparities 2021a, 2021b, 2021c, 2024). This issue appears to be worst in the North West (9.9% vs. national average in England of 8.5% of adults), with the North East (8.8%) and Yorkshire and the Humber (8.6%) regions showing slightly lower prevalence. Type II diabetes prevalence in adults (aged 16y or above) is also slightly higher in the three regions of the North (8.2% in the North East, *0.1% in the North West and 8.0% in Yorkshire and the Humber respectively) than the national average (7.8%) (Office for Health Improvement and Disparities 2024).

### 2.2 The North Appears to Have Poorer Diet Quality and Food Provision Than the Rest of England

Data from the Global Burden of Disease database (Global Burden of Disease Collaborative Network 2020) indicate that the dietary intake of calcium, fibre, vegetables, fruit, legumes, and sodium is all generally worse than the national average in the North of England. However, there are major limitations in data on current, regional dietary habits across England/the UK, particularly in comparison to available data on health outcomes. The UK's existing national dataset, the National Diet and Nutrition Survey (NDNS), is not adequately scaled for investigation of region‐specific variations in intake. Since 2012, NDNS has aimed to collect nationally representative data from a relatively small cohort of c.500 participants (Public Health England 2020). These data also broadly underline sub‐optimal national dietary habits, with a modest association of higher household income and more positive dietary habits (Public Health England 2020). A dataset of this size does not allow regional sub‐analysis with any statistical certainty, although previous approaches suggest deficiencies in overall diet quality in the North of England, particularly in relation to the prevalence of fruit and vegetable consumption below one serving per day (Smith et al. 2021).

Collated data on dietary risk factors from the Global Burden of Disease database highlight that core aspects of dietary habits linked to national plans for health improvement appear to be among the consistently worst in England. However, those in the North have dietary habits worse than the English average in terms of intake of calcium, fibre, sodium (notably only for Yorkshire and The Humber region), legumes, and polyunsaturated fatty acids.

Taken as a whole, the above dietary pattern would predispose populations to be at greater risk of cardiovascular, metabolic (including obesity) and musculoskeletal disease. Such conditions have major healthcare and wider societal costs. Healthcare costs of ill health as a result of poor diet and excess body weight have been previously estimated to account for over 10% of the total NHS budget (Scarborough et al. 2011). Recent estimates suggested the total economic and societal costs of obesity to be £126 billion in 2025 alone (Nesta 2025). Many of the above elements of sub‐optimal diet stand in direct contrast to national guidelines, which recommend limiting intake of foods high in fat, salt and sugar while also targeting increased intake of fibre (Public Health England 2016a, 2016b). The available data also suggest that not all aspects of dietary habits in the North of England are worse than the national average but also underline the need for regionally targeted interventions (Smith et al. 2021).

The North East has the highest density of fast food outlets per 100 000 people (106.7) for any region in England (average 81.9 outlets/100000 people), with the North West (104.7 outlets/100000 people) and Yorkshire and the Humber (99.5 outlets/100000 people) also falling within the worst quartile of the country. Data collected in 2014 also highlight the high density of fast food outlets across the three regions of the North, although Yorkshire and the Humber had the greatest density within this dataset (Office for Health Improvement and Disparities 2024).

### 2.3 The North of England Has Greater Adult and Child Food Insecurity

This is represented most directly through lower average weekly household food expenditure in all three regions in The North. Data on unadjusted household expenditure from 2020 to 2022 (Office for National Statistics 2023d) suggest that the absolute amount spent weekly on food and non‐alcoholic drinks is less in the three regions of the North of England than the national average (£65.50), particularly in the North East (£56.30) and Yorkshire and the Humber (£58.20), with lower absolute expenditures across core food groups like starchy staples, fruit and vegetables, and dairy products. The proportion of expenditure on fruit and vegetables is also somewhat lower (North East 19.6%, North West 20.0%, Yorkshire and the Humber 21.3% versus England average of 22.4%), which equates to between £1.67 and £3.27 less absolute expenditure per week (Office for National Statistics 2023d). Office for Health Improvement and Disparities data also highlight that the North West has the highest percentage of households experiencing food insecurity (13%—as measured by the food security questions in the Family Resources Survey) in the whole of England (Office for Health Improvement and Disparities 2021a, 2021b, 2021c, 2024). Further detail on the calculation of these household expenditure estimates is available through the Office for National Statistics website (Office for National Statistics 2023c).

Free school meal eligibility is often used as a proxy of lower household income. The regions of the North have the highest proportion of eligibility across the whole of the UK, underlining previous reports of increased risk of economic hardship (Health Equity North 2023). The North East has the highest eligibility (30.4%), with similar levels in the North West (26.8%) and Yorkshire and the Humber (26.0%) (Office for Health Improvement and Disparities 2024). Together, the above findings highlight that a number of embedded challenges exist across the North in relation to access or availability of positive food choices.

## 3 Examples of Place‐Based Research Within the North of England

Within the report, we presented five research case studies of nutrition‐related success from across the wider Nutrition North Consortium. These are examples of a few of the impactful projects and expertise that exist in the multidisciplinary area of nutritional research across the North of England.

### 3.1 The Wider Food Environment

Recent work has involved application of expertise in qualitative and quantitative observational studies to better understand the evolving food environment. Advertising of unhealthy food and drink detrimentally influences children's diets and contributes to the development of obesity (Boyland et al. 2022; Norman et al. 2016). The North East of England contains some of the most deprived local authorities in the UK (Ministry of Housing Communities and Local Government 2025), with some of the highest prevalences of childhood obesity (NHS England 2023). Local authorities are responsible for the control of advertising on council‐owned spaces and are increasingly implementing restrictions to advertising on their estates to support public health.

Researchers explored the extent and nature of bus shelter advertising in Middlesborough, as well as Redcar and Cleveland (Finlay et al. 2022). This research identified that over half of all bus shelter advertisements were for food, with over a third of all food ads classed as ‘less healthy’ by the existing UK Nutrient Profiling Model. Frequent products advertised were sugar sweetened beverages (35%), burgers/sandwiches (24%) and French fries (21%). Persuasive strategies that appeal to young people, such as food images and competitions, were frequently observed in the promotion of less healthy foods.

The findings of this work have been disseminated through blog posts for UK‐based charities, non‐governmental organisations (NGOs) and local radio. Additionally, this evidence has been used by NGOs and charities operating in the health space to support their campaigns and activities to drive healthier food environments. Middlesborough council have recently (April 2026) announced a healthier advertising policy on council‐owned sites (Middlesbrough Council 2026). Once implemented, this research can be used as a baseline measure to assess the effectiveness of restrictions in reducing the prevalence of less healthy food advertising in outdoor environments.

### 3.2 Research Co‐Designed With Key Stakeholders

A Sport England–funded project conducted in two of the most deprived local authorities in the North East of England explored how adults with type 2 diabetes engaged with remission‐focused diet and physical activity strategies (Boocock et al. 2024). The study demonstrated that adherence to prescribed dietary approaches could lead to improvements in glycaemic control and body weight. However, patient narratives revealed important limitations within routine diabetes care. While flexibility, choice, and opportunities to integrate change into everyday life supported engagement, experiences of stigma, judgement, and dietary advice perceived as unrealistic, within constrained socioeconomic circumstances, often undermined adherence.

These findings prompted a shift towards examining the wider conditions shaping dietary adherence, particularly food insecurity. Subsequent work by the Teesside University research team synthesised evidence examining how food insecurity is identified and addressed within type 2 diabetes care (Creevy and Boocock 2026). Although clinicians recognised food insecurity as relevant to diabetes outcomes, it remained largely invisible in routine care. Screening was rarely embedded, constrained by time pressures, limited organisational ownership and the absence of clear, system‐led referral pathways. The review also highlighted the importance of trust‐based, non‐judgemental clinical relationships in enabling disclosure, alongside the limits of clinical care in the absence of effective links to community and voluntary sector support.

Together, this work supports a systems‐thinking approach that integrates organisational reform, relational care and stronger clinical–community partnerships. Ongoing research aims to develop and test NHS‐ready pathways to identify and respond to food insecurity within routine diabetes services.

Good nutrition during the first 1000 days of life (from conception to a child's second birthday) has a profound and lasting impact on health across the lifespan. UK Antenatal Care Guidelines recommend that midwives discuss nutrition with women at booking appointments. However, nutrition often receives low priority in practice due to midwives' limited formal training in this area. Consequently, many pregnant women report that the nutrition advice they receive is non‐existent, vague, or inconsistent. Strengthening midwives' confidence and competence in delivering nutrition advice is therefore urgently needed to improve maternal and infant health outcomes.

To address these gaps, several educational initiatives have been developed for both student and qualified midwives. A dedicated chapter on Nutrition in Healthy Pregnancy was authored and included in the 2023 edition of the core midwifery textbook Mayes' Midwifery. In addition, a structured nutrition curriculum for student midwives was designed and delivered at Edge Hill University, Liverpool John Moores University, and the University of Manchester. Nutrition training for qualified midwives was supported through study days held in Manchester, Leeds, and Sheffield.

Further professional engagement was achieved through healthcare‐focused podcasts, achieving over 18 500 listens. Feedback from students indicates increased confidence in delivering nutritional messages, greater appreciation of the importance of nutrition, and a clear intention to prioritise nutrition discussions in future midwifery practice (Charnley et al. 2024; Stone et al. 2024).

### 3.3 Policy‐Facing Research and Partnerships

Local authorities have had responsibility for public health which includes promoting a healthy environment since 2012. To help them achieve this objective, over half of all local authorities have implemented some type of supplementary planning document to restrict the proliferation of new hot food takeaways (Keeble et al. 2019). However, the lack of evidence of efficacy of such approaches limited the potential for public health teams to justify such approaches at council/local authority level and would increase the risk of rulings against businesses being legally contested.

Research involving partner organisations from the North West and North East evaluated the impacts of recent, multipronged approaches by Gateshead council to limit childhood obesity rates through town planning. This research presented initial evidence that town‐planning policies could have impacts on population‐level childhood body weight status outcomes and limited the potential for future the policy to be challenged by businesses because of a lack of evidence on effectiveness. This work filled this important policy gap with the first peer‐reviewed published research to provide evidence on the effectiveness of this type of policy on population health. The findings from our research are being used by local and national government to develop regional policy.

At the local authority level, the evidence generated on the effectiveness of Gateshead's Supplementary Planning Document restricting hot food takeaways is currently being used by Gateshead Council to develop their local plan. This evidence informed the development of supplementary planning guidance restricting hot food takeaways in Lancashire (Lancashire County Council 2024). These findings have also been used by Sheffield City Council as evidence to refuse planning permission for a McDonalds near a primary school (Wilkinson 2026). This highlights how the policy has been used to implement their policy on promoting a healthy food environment.

Food Active's Healthy Weight Declaration (HWD) was developed in consultation with stakeholders across the North West, including Directors of Public Health and academic partners, to support local government in adopting a whole‐systems approach to promoting healthy weight. Since its introduction, it has been used by a wide range of local authorities (county, unitary, metropolitan and district councils) to strengthen system leadership in policymaking and improve opportunities for communities to live healthier lives. More recently, the Declaration has evolved into a broader framework that enables collaboration among partners at place level.

The HWD is a formal statement owned by each adopting authority, signalling a strategic, organisation‐wide commitment to reducing unhealthy weight, protecting the health and wellbeing of staff and residents, and generating positive impacts on health, social care and the local economy. It has been adopted by more than 36 councils across England, including 23 in the North. Emerging evidence suggests it is an effective tool for bringing stakeholders together, raising the profile of healthy weight, and shaping local policy agendas.

A recent stakeholder evaluation highlights several key themes. First, its strategic value lies in providing a shared framework and common language that elevates healthy weight as a corporate priority beyond public health. Second, in terms of leadership and governance, adoption has secured senior political and executive buy‐in, often embedded within Health and Wellbeing Boards. Finally, the Declaration has influenced policy and system change, particularly in planning, advertising, procurement and commissioning, supporting stronger cross‐departmental collaboration (Food Active 2026).

While the report focused on a limited number of research case studies, there are many other examples of the outstanding nutrition research occurring across the Nutrition North Consortium and more widely across the region. These previous research successes include research informed by all disciplines that inform human nutrition. The work has included sociological, analytical, systems‐based, foundational, public health, and clinically‐focused research representative of breadth and depth of nutrition expertise in the North of England. A previous report from NHSA noted inequity in (clinically‐related) research between the North and the South (Martyn 2024), further underlining the challenge ahead. Nutrition North's future work will leverage the existing research excellence in the North of England to benefit diet and health regionally, nationally, and globally.

## 4 Conclusions

There appear to be major, existing, nutritional challenges in the three regions of the North, particularly dietary habits, food access and more frequent diet‐related health issues. These problems are considerably worse than the already far from ideal norms seen across the rest of England. Specific areas (i.e., by council or unitary authority) of The North appear to fare better than the rest of England or their immediate geographical counterparts. Further considerations of commonalities in these areas could support development of a blueprint for change in areas with worse health outcomes or dietary habits.

The core recommendations of the Nutrition North report are presented below. It must be noted that evidence of efficacy of specific actions is limited globally in, for example, public health interventions to manage excess body weight (Eskandari et al. 2022; Ruxton et al. 2025), dietary habits (Pineda et al. 2022) and health inequalities. As such, there is an immediate need for bold, long‐term, multi‐pronged and forward‐thinking action to limit the commonality of geographically‐related nutritional inequalities evidenced above. Longer‐term planning should focus on research/evaluation of the success, cost‐effectiveness and applicability of interventions. The Nutrition North report made the following recommendations with this in mind and based on current or on‐going activities.

1. Improve dietary monitoring at a regional level—granular data in alignment with national, region‐specific health data would allow more targeted efforts from Local Authorities. Recent changes to the NDNS, including greater use of online data collection methods, may facilitate the collection of a broader range of dietary and health information in the future (Office for Health Improvement and Disparities 2025). Expanding the total number of respondents to a national survey would provide opportunities for more detailed and timely monitoring of dietary patterns and nutritional status across the UK population.
2. Develop and implement strategies to improve access to positive food choices in the North—the research case studies included in the report are positive templates that can guide future approaches in the North of England. Due to the urgent need for change at a regional level, we propose utilisation of the existing policy options available to Local Authorities. This includes:
  - Embedding a skilled nutritionist workforce in the three regions of the North, as suggested in the National Food Strategy (Department for Environment Food and Rural Affairs 2022)
  - Expanding voucher schemes like The Healthy Start scheme (Barrett et al. 2024), informed by regional needs, to make positive food choices more economically realistic.
  - Auto‐enrolment onto Free School Meals and equivalent*—*Free School Meals/Universal Free School Meals provide meals of a minimal legal nutritional standard to children of primary and secondary school age (Department for Education 2024). Auto‐enrolment for free school meals is currently being trialled by Middlesbrough Council (2024). Wider auto‐enrolment may represent a way of supporting increased uptake to embed positive eating habits and limit risk of child food insecurity. Similarly, recent approval of longer‐term funding for national Holiday Activity and Food programmes provides opportunity for similar nutrition (and activity) support outside of the school term (UK Parliament 2025).
  - Deliver targeted food assistance/support schemes*—*issues of food access appear to more frequently affect the people of the North (Office for Health Improvement and Disparities 2024; Office for National Statistics 2023d). Food banks, food larders, and social supermarkets all provide a route to improve food access if properly funded at a regional and national level.
  - Expansion of ‘High Fat, Salt and Sugar Food (HFSS) Ad Bans’ at a Local Authority level*—*specific local authorities in the North of England have or are aiming to restrict public routes of advertising (e.g., within bus shelters). While challenging, altering ingrained perceptions of norms around food has the potential to change dietary habits over time. Since writing the original Nutrition North report, national restrictions on advertising of foods high in fat, salt and sugar have come into force (Department of Health and Social Care 2025).
  - Supporting Local Authorities Development of Healthy Planning guidance—many local authorities in England have driven health promotion through Supplementary Planning Documents (SPD), including those in the North, with some evidence of impacts on childhood obesity rates (Xiang et al. 2024). Evaluating and supporting best practice across multiple local authorities will benefit future strategy in the North and further afield.

Nutrition North will work with the wider Northern Health Science Alliance partnership, local authorities and wider stakeholders (e.g., school food caterers, food assistance scheme organisers) to ensure nutrition research in the region leads to real‐world impact. To support the growth of nutrition in the North of England, we aim to expand our membership to ensure representation across all three regions. We will also target collaborative funding to further develop the existing network.

## Author Contributions

All authors (I.B., J.A., R.B., E.B., H.B., N.C., A.F., M.G., S.L., A.A.L., S.H. and B.M.C.) developed the final report collaboratively. I.B. drafted the initial version, which was conceptualised by B.M.C. and A.A.L. All authors (I.B., J.A., R.B., E.B., H.B., N.C., A.F., M.G., S.L., A.A.L., S.H. and B.M.C.) checked and agreed revised drafts of the manuscript and agreed on the final submission version. AI has not been used to in the preparation of this manuscript.

## Funding

The authors have nothing to report.

## Conflicts of Interest

The authors declare no conflicts of interest.

## Data Availability

Data sharing not applicable to this article as no datasets were generated or analysed.
